# Supplementary material for: TRPA1 activation in non-sensory supporting cells contributes to regulation of cochlear sensitivity after acoustic trauma
Source: Nat Commun. 2023 Jun 30;14:3871. doi: 10.1038/s41467-023-39589-w (PMC10313773; doi:10.1038/s41467-023-39589-w)
Supplement: Supplementary file 1 — Supplementary Information [file 41467_2023_39589_MOESM1_ESM.pdf]

Supplementary Information for

TRPA1 activation in non-sensory supporting cells contributes to regulation of cochlear sensitivity after acoustic trauma

A. Catalina Vélez-Ortega[1,*], Ruben Stepanyan[1,2], Stephanie E. Edelmann[1], Sara Torres-Gallego[1], Channy Park[3], Desislava A. Marinkova[1,4], Joshua S. Nowacki[1], Ghanshyam P. Sinha[1,5], Gregory I. Frolenkov[1,*]

[1] Department of Physiology, College of Medicine, University of Kentucky, Lexington, KY 40536 USA

[2] Current address: Department of Otolaryngology, Case Western Reserve University, Cleveland, OH 44106 USA

[3] Department of Head & Neck Surgery, David Geffen School of Medicine, UCLA, Los Angeles, CA 90095 USA

[4] Current address: Department of Pharmacology and Toxicology, University of Arkansas for Medical Sciences, Little Rock, AR 72205 USA

[5] Current address: Department of Anesthesiology and Perioperative Medicine, University of Pittsburgh, Pittsburgh, PA 15261 USA

*Corresponding authors: A. Catalina Vélez-Ortega (catavelezo@uky.edu), Gregory I. Frolenkov (Gregory.Frolenkov@uky.edu).

Supplementary Figures:

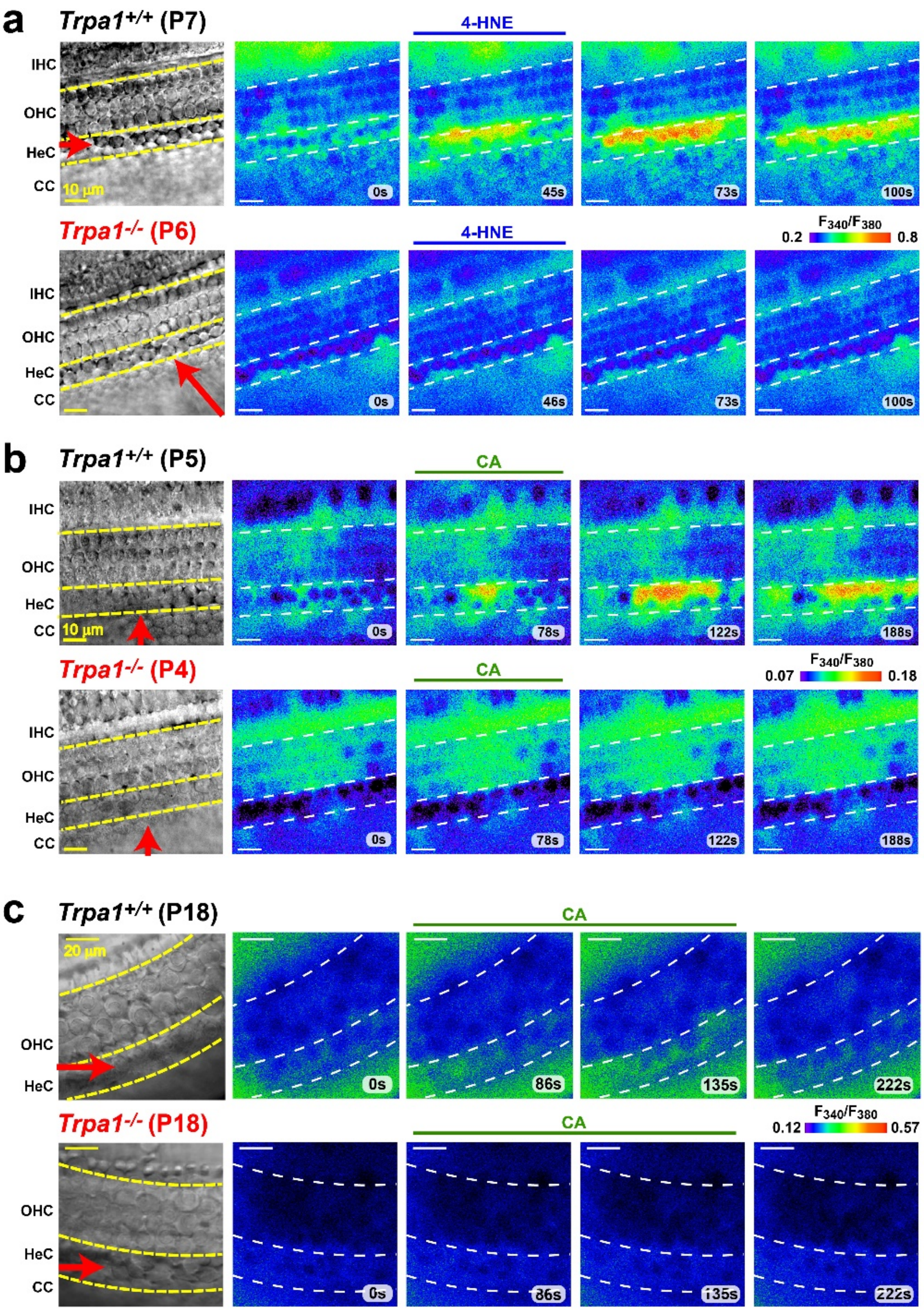

Supplementary Figure 1. ***Long-lasting Ca^{2+} responses in Hensen's cells evoked by the application of TRPA1 agonists.***

(a,b,c) Ratiometric (F_{340}/F_{380}) fura-2 images without normalization to the average pre-stimulus background for the time-lapse frames presented in Fig.2a,d,g. Layout of the panels is identical to Fig.2a,d,g. F_{340}/F_{380} ratio is proportional to the absolute concentration of free cytosolic Ca^{2+}. Source data are provided as a Source Data file.

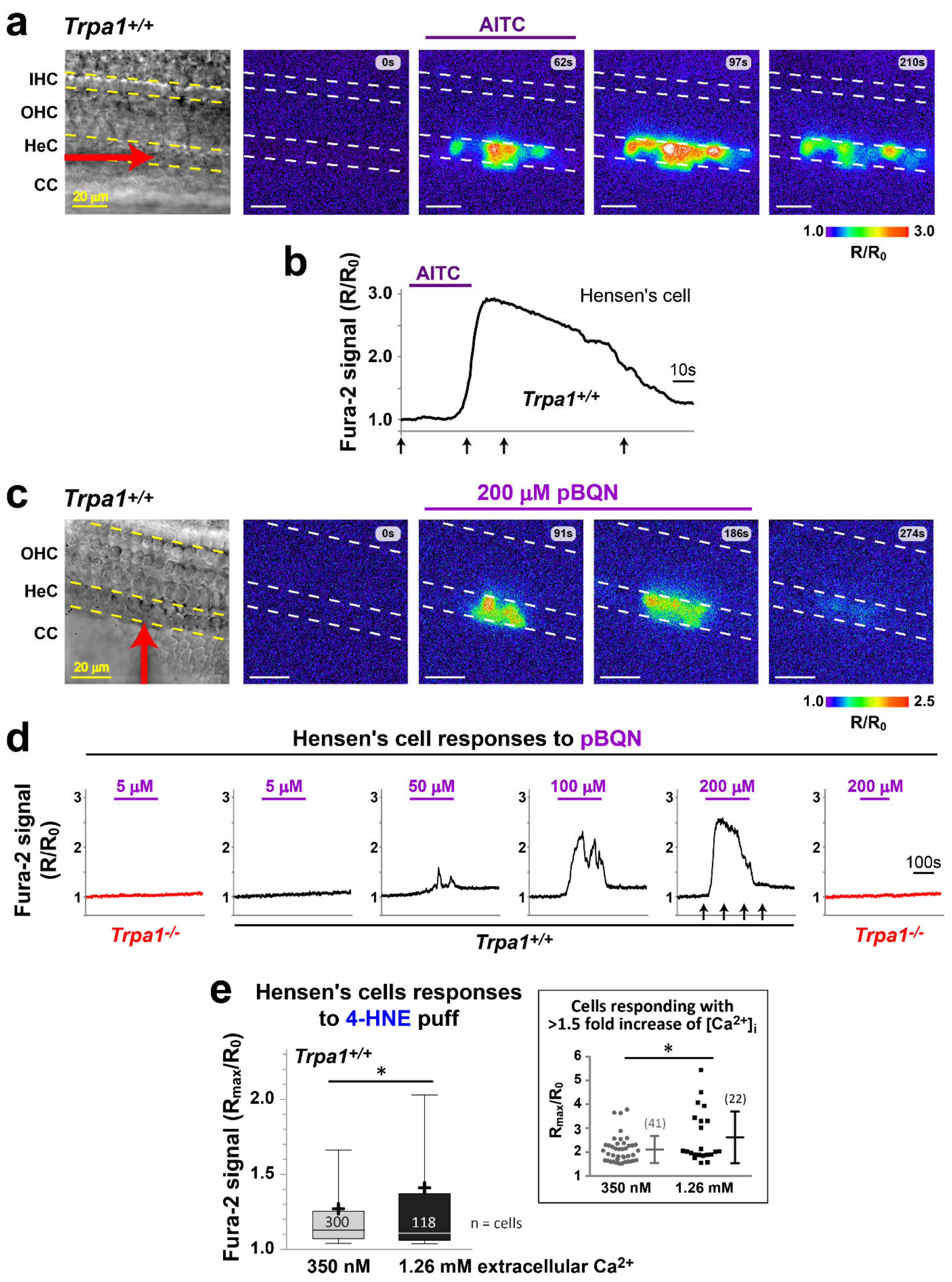

Supplementary Figure 2. ***TRPA1-mediated Ca^{2+} responses in Hensen's cells to puff applications of AITC and pBQN.***

(a) Time-lapse imaging of Ca^{2+} responses to the application of mustard oil (AITC, 100 µM) in a cochlear explant from a wild-type P1 mouse. The reference bright field image indicating the position of the puff pipette (red arrow) is on the left, while other frames show the $R = F_{340}/F_{380}$ ratio of fura-2 signals in pseudocolor

scale normalized to the pre-stimulus baseline (R_0). Images are representative of 4 independent experiments. **(b)** Representative Ca^{2+} response in one of the Hensen's cells shown in **a**. Arrows at x-axis indicate the timing of frames in panel **a**. **(c)** Time-lapse imaging of Ca^{2+} responses to the local application of 200 μM *para*-benzoquinone (pBQN) in a cochlear explant from a P6 wild-type mouse. Layout of the panel is identical to **a**. **(d)** Representative Ca^{2+} responses to varying concentrations of pBQN in wild-type (black) and *Trpa1*$^{-/-}$ (red) Hensen's cells. Arrows at the x-axis of the Ca^{2+} response to 200 μM benzoquinone indicate the timing of the frames in **c**. Images are representative of 3 independent experiments. **(e)** Normalized amplitude (R_{max}/R_0) of Ca^{2+} responses to 200 μM 4-HNE in wild-type Hensen's cells in the extracellular medium containing either 350 nM or 1.26 mM Ca^{2+}. Each boxplot shows the median (center line), 25th and 75th percentile (box limits), the 10th and 90th percentile (whiskers), and the mean as '+'. The asterisk indicates statistical significance (P=0.015, two-sided Student's *t* test). The inset shows the subset of these data from the cells responding with at least a 1.5 fold increase of $[Ca^{2+}]_i$, and the error bars indicate Mean±SD. The asterisk indicates statistical significance (P=0.018, two-sided Student's *t* test). Source data are provided as a Source Data file.

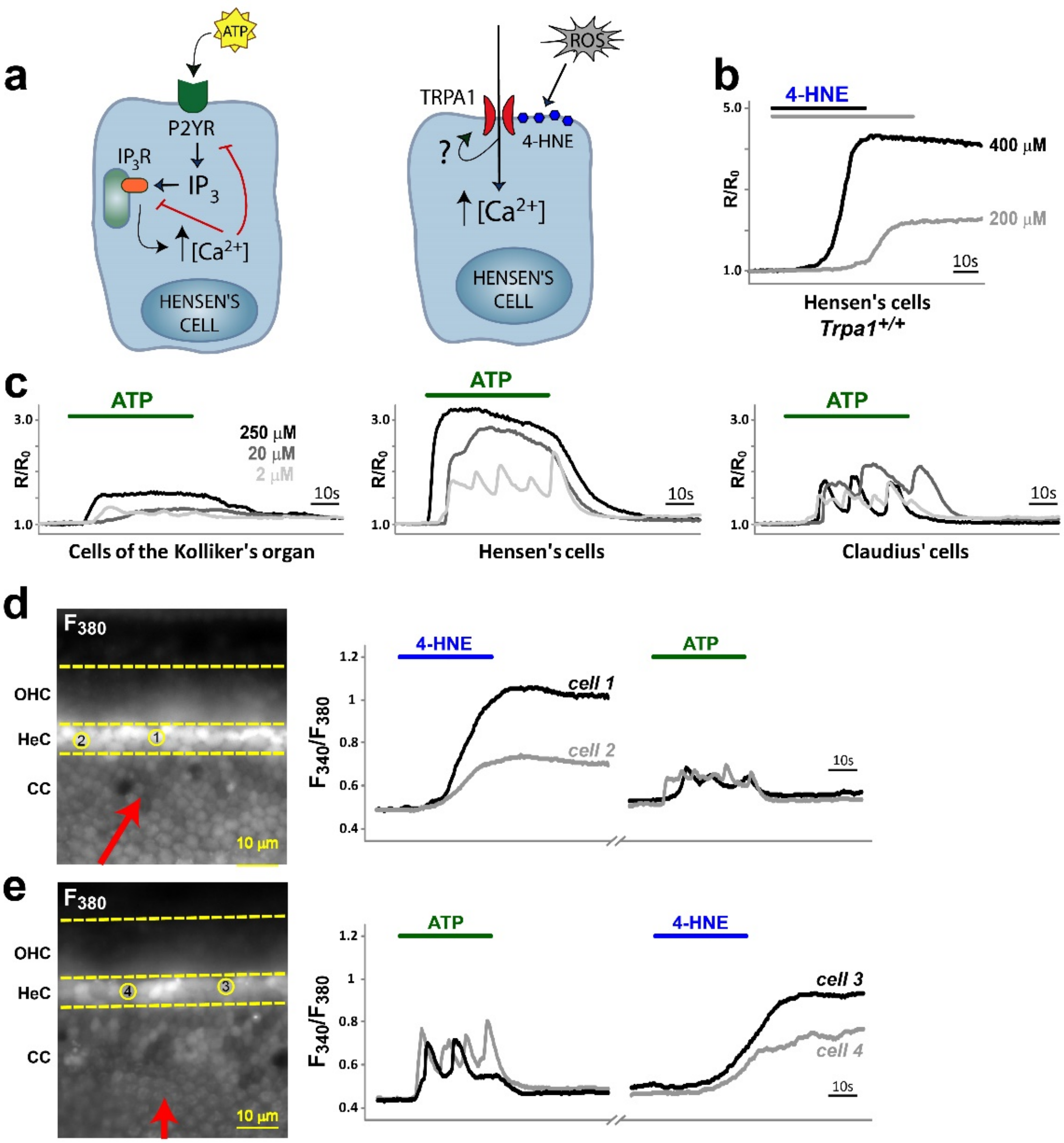

Supplementary Figure 3. ***Different kinetics of Ca^{2+} responses evoked by extracellular ATP and TRPA1 agonists in supporting cells of wild-type mice.***

(a) Potential mechanisms of Ca^{2+} responses to extracellular ATP (*left*) and TRPA1 agonists (*right*). *Left*, at low ATP concentrations, oscillating Ca^{2+} responses have been associated with activation of G-protein-coupled ATP receptors (P2YR) and their subsequent desensitization, as well as with the Ca^{2+}-dependent feedback inhibition of Ca^{2+} release from the intracellular stores. *Right*, a TRPA1 channel may be 'locked' in an open state due to covalent modifications of TRPA1 by a reactive agonist (*e.g*. 4-HNE) or due to the potentiation of TRPA1 activation by intracellular Ca^{2+}. **(b)** Representative Ca^{2+} responses to the puff application of 200 μM (gray) and 400 μM (black) of 4-HNE in Hensen's cells. Note that even the decreased response to 4-HNE at 200 μM does not show oscillations and continue long after the end of agonist application. **(c)** Representative Ca^{2+} responses to the application of 2, 20 and 250 μM ATP (darker traces indicate higher ATP concentrations) in Kolliker's organ (*left*), Hensen's cells (*middle*) and Claudius' cells (*right*). **(d,e)** Ca^{2+} responses in two Hensen's cells (black and grey) of a wild-type mouse evoked by the application of 4-HNE (200 μM) followed by ATP (2 μM) (**d**) or vice versa (**e**). Left panels show F_{380} images of the analyzed cells (circled numbers) and positioning of the puff pipettes (red arrows). Breaks in the time axes on the right graphs represent the washout periods after the first stimulation. During this washout, the puff pipette was carefully replaced to deliver a new drug from the same position. Notice that the same cells exhibited oscillating short-lived Ca^{2+} responses to ATP but long-lasting Ca^{2+} responses to 4-HNE, regardless of the order in which these stimuli were delivered. The data are representative of 3 independent series. Source data are provided as a Source Data file.

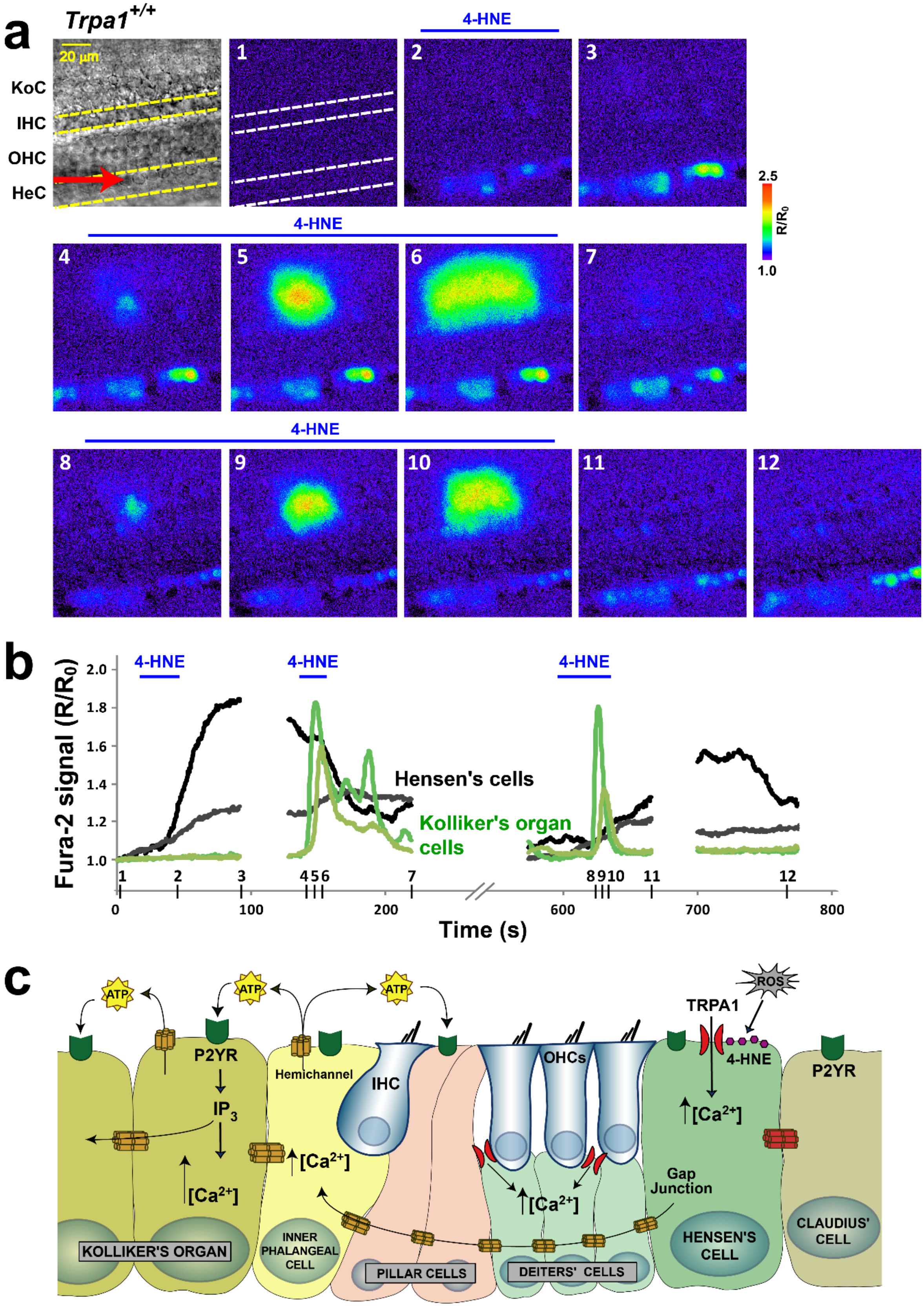

Supplementary Figure 4. ***TRPA1-initiated Ca^{2+} responses propagate from Hensen's cells toward the Kolliker's organ at "hot spots".***

(a) Consecutive applications of 400 µM of 4-HNE to a Hensen's cell region evoke Ca^{2+} responses propagating to the Kolliker's organ. First panel shows a reference bright field image with the puff pipette position (red arrow). Consecutive 4-HNE applications are indicated with blue horizontal bars above the

images. Notice that the second and third stimulations of Hensen's cells led to the propagation of Ca^{2+} responses to the same area of the Kolliker's organ. Data are representative from 7 independent experiments where the propagation of Ca^{2+} responses was observed. **(b)** Changes of $[Ca^{2+}]_i$ in two Hensen's cells (black and gray) and two cells of the Kolliker's organ (shades of green) from the experiment shown in **a**. The numbered ticks at the x-axis indicate the timing of frames in **a**. **(c)** TRPA1-initiated signals in the organ of Corti. On the endolymphatic side, TRPA1 channels in Hensen's cells are the first to respond to endogenous byproducts of oxidative stress such as 4-HNE. Long-lasting Ca^{2+} responses in Hensen's cells do not activate adjacent Claudius' cells but, instead, propagate across the organ of Corti to the Kolliker's organ. This propagation most likely involves the gap-junctional conductance through Deiters' and pillar cells. Rise of intracellular Ca^{2+} can initiate ATP release to the extracellular space and trigger Ca^{2+} waves in the Kolliker's organ. These 'secondary' Ca^{2+} waves in the Kolliker's organ depend on binding of extracellular ATP to P2Y receptors, thus resembling the Ca^{2+} waves occurring after OHC damage. TRPA1 channels in Deiters' cells may sense byproducts of oxidative stress in the perilymph. Source data are provided as a Source Data file.

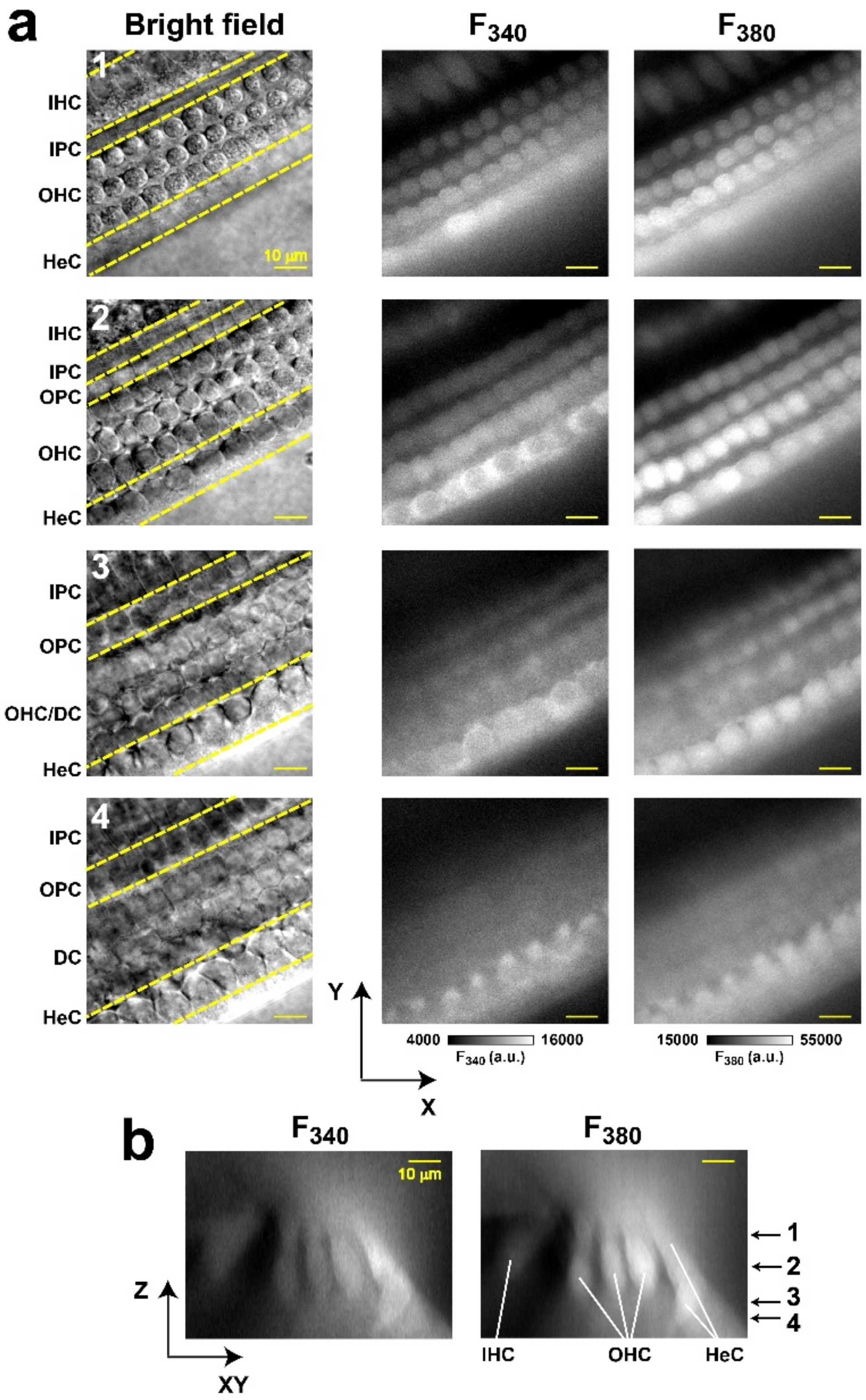

Supplementary Figure 5. ***Non-uniform loading of the cochlear epithelium with membrane-permeable fura-2.***

(a) *Z*-stack of bright-field (*left*) and fura-2 images in a cochlear explant obtained with the 340 (*middle*) and 380 (*right*) nm illumination. Reference bright-field images show the boundaries between different cell types in the cochlear epithelium. **(b)** Orthogonal sections of fura-2 fluorescence reconstructed from the data shown in **a**. The arrows on the far-right side indicate the focal planes of the images shown in **a**. Notice the lack of fluorescence in both channels (F_{340} and F_{380}) in the pillar cell area between the inner and outer hair cells and in Deiters' cells below OHCs. Data are representative of over 50 independent experiments. Source data are provided as a Source Data file.

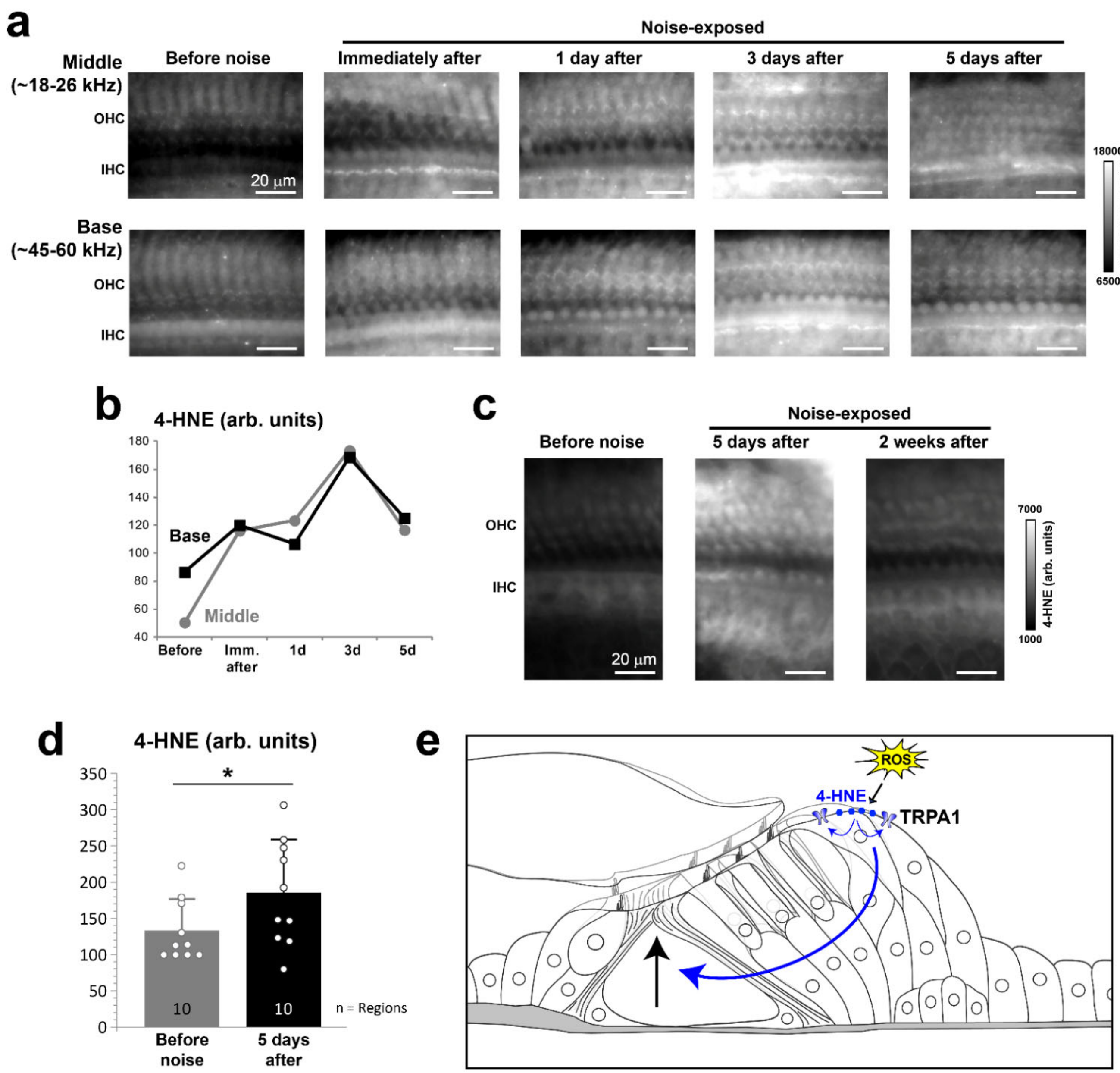

Supplementary Figure 6. ***Delayed increase of 4-HNE-modified proteins following noise exposure.*** **(a)** Immunolabeling of 4-HNE-modified proteins in the organ of Corti from the middle (*top*) and basal (*bottom*) cochlear turns from 4-week-old mice before and at several time points after a single exposure to wide-band noise at 100 dB SPL for 30 min. Data are representative of 3 independent experiments. **(b)** Quantification of the 4-HNE fluorescence (arb. units, arbitrary units) from the panels shown in **a**, indicating the progressive increase of oxidative stress byproducts throughout several days after noise exposure. **(c)** Immunolabeling of 4-HNE-modified proteins in the organ of Corti of 3-week-old mice before (*left*), 5 days after (*middle*), and 2 weeks after (*right*) noise exposure, showing the eventual decrease of 4-HNE production by the second week after the acoustic trauma. Each image represents the average of a Z-stack covering the thickness of the organ of Corti. Data are representative of 3 independent experiments. **(d)** Quantification of 4-HNE labeling before and 5 days after noise exposure in three independent series (two exposed to 100 dB SPL for 30 min and one to 110 dB SPL for 2 hours). Data are shown as Mean±SE, and the asterisk indicates statistical significance (P=0.049, paired two-sided Student's *t* test). **(e)** For several days after the noise exposure, the increase in reactive oxygen species (ROS) generates byproducts, such as 4-HNE, that can activate TRPA1 channels in the Hensen's cells. Then, Ca^{2+} signals propagate to Deiters' and pillar cells. The combined TPA1-initiated changes in the shapes of all these supporting cells could modify the geometry of the organ of Corti and elevate hearing thresholds for days after the noise exposure. Source data are provided as a Source Data file.

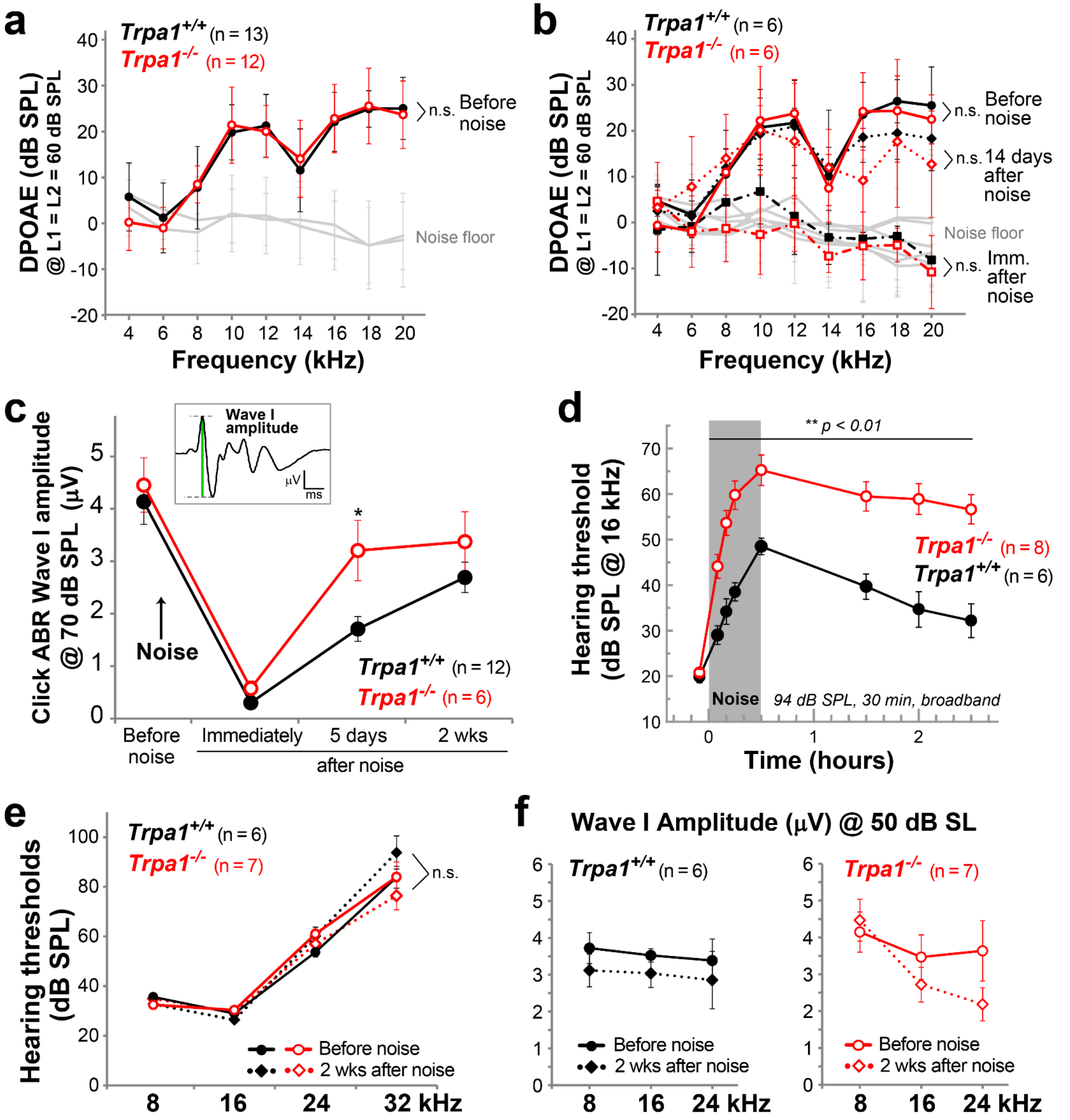

Supplementary Figure 7. ***Further characterization of noise effects on cochlear function in wild-type and $Trpa1^{-/-}$ mice.***

(a) Baseline distortion product otoacoustic emissions (DPOAE) at different frequencies in wild-type (black, filled symbols) and *$Trpa1^{-/-}$* mice (red, open circles) before noise exposure. **(b)** In a subset of experiments from panel (a), DPOAEs were measured before, immediately after (squares, dash-dot lines), or 14 days after (diamonds, dotted lines) exposure to moderate broadband noise (100 dB SPL, 30 min). **(c)** Amplitude of click-evoked ABR Wave I (as indicated in cartoon) in wild-type (filled symbols) and *$Trpa1^{-/-}$* (open symbols) mice after moderate noise exposure (100 dB SPL for 30 min). **(d)** Hearing thresholds in wild-type (black, filled symbols) and *$Trpa1^{-/-}$* (red, open symbols) mice determined with 16 kHz tone burst-evoked auditory brainstem responses (ABR) at several time points during and after exposure to moderate broadband noise (94 dB SPL, 30 min). **(e)** Thresholds of tone burst-evoked ABRs in wild-type (black, filled

symbols) and *Trpa1*$^{-/-}$ (red, open symbols) mice before (circles, solid lines) and 2 weeks after (diamonds, dotted lines) exposure to mild broadband noise (85 dB SPL, 30 min). **(f)** Amplitudes of tone burst-evoked ABR Wave I in wild-type (filled symbols) and *Trpa1*$^{-/-}$ (open symbols) mice before (circles, solid lines) and 2 weeks after (diamonds, dotted lines) exposure to mild (85 dB SPL for 30 min) broadband noise in the same mice that are shown in Fig.8e. Data are shown as Mean ± SD (**a**,**b**) or Mean ± SE (**c**,**d**,**e**,**f**). Asterisks indicate statistical significance between genotypes (∗, P<0.05; ∗∗, P<0.01) determined either at specific data points by two-sided Student's *t* test (**c**,**d**) or by two-way ANOVA between grouped data (**a**,**b**,**e**,**f**); *n.s.* not significant. In panel **c**, P=0.017 at 5 days after noise in panel; in panel **d**, P=0.003, 0.0004, 0.0003, 0.002, 0.001, 0.0008 and 0.0005 for 5, 10, 15, 30, 90, 120 and 150 minutes during/after noise exposure. Source data are provided as a Source Data file.

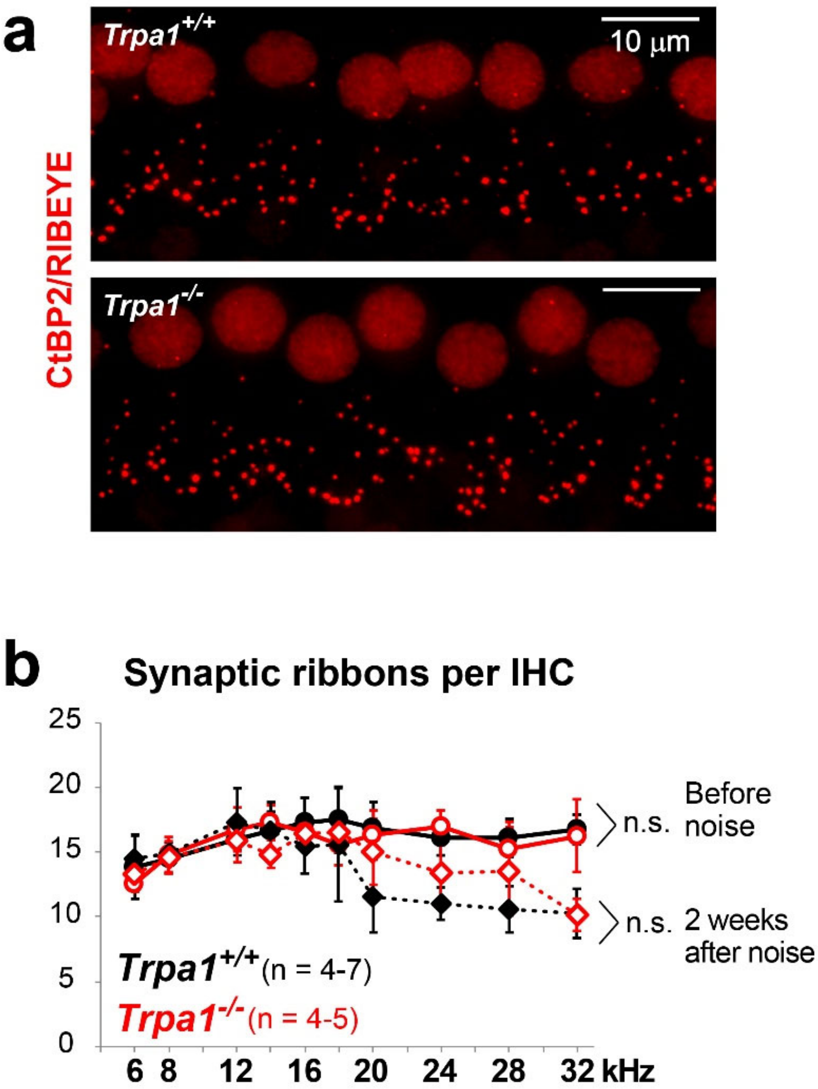

Supplementary Figure 8. ***TRPA1 deficiency does not exacerbate noise-induced loss of IHC ribbon synapses at high frequencies.***
(a) Representative maximum-intensity projections of CtBP2/RIBEYE immunolabeling (red) in IHC from wild-type (*top*) and *Trpa1*$^{-/-}$ (*bottom*) mice at the 16 kHz cochlear region before noise exposure. **(b)** IHC synaptic ribbon counts along the cochlear length in wild-type (black) and *Trpa1*$^{-/-}$ (red) mice, before (circles, continuous lines) and two weeks after (diamonds, dotted lines) exposure to moderate 100 dB SPL broadband noise for 30 min. The differences between genotypes were not significant (before noise: P=0.399, n=4 mice in each group; after noise: P=0.182, n=7 and n=5 mice in wild-type and *Trpa1*$^{-/-}$ groups, correspondingly; two-way ANOVA). Data are shown as Mean±SD (n.s. not significant). Source data are provided as a Source Data file.
