# Supplementary material for: TRPA1 activation in non-sensory supporting cells contributes to regulation of cochlear sensitivity after acoustic trauma
Source: Nat Commun. 2023 Jun 30;14:3871. doi: 10.1038/s41467-023-39589-w (PMC10313773; doi:10.1038/s41467-023-39589-w)
Supplement: Supplementary file 8 — Reporting Summary [file 41467_2023_39589_MOESM8_ESM.pdf]

nature portfolio

Corresponding author(s): Gregory Frolenkov & A. Catalina Vélez-Ortega

Last updated by author(s): May 5 2023

Reporting Summary

Nature Portfolio wishes to improve the reproducibility of the work that we publish. This form provides structure for consistency and transparency in reporting. For further information on Nature Portfolio policies, see our Editorial Policies and the Editorial Policy Checklist.

Statistics

For all statistical analyses, confirm that the following items are present in the figure legend, table legend, main text, or Methods section.

n/a	Confirmed	
☐	☒	The exact sample size (*n*) for each experimental group/condition, given as a discrete number and unit of measurement
☐	☒	A statement on whether measurements were taken from distinct samples or whether the same sample was measured repeatedly
☐	☒	The statistical test(s) used AND whether they are one- or two-sided<br>*Only common tests should be described solely by name; describe more complex techniques in the Methods section.*
☒	☐	A description of all covariates tested
☐	☒	A description of any assumptions or corrections, such as tests of normality and adjustment for multiple comparisons
☐	☒	A full description of the statistical parameters including central tendency (e.g. means) or other basic estimates (e.g. regression coefficient) AND variation (e.g. standard deviation) or associated estimates of uncertainty (e.g. confidence intervals)
☐	☒	For null hypothesis testing, the test statistic (e.g. *F*, *t*, *r*) with confidence intervals, effect sizes, degrees of freedom and *P* value noted<br>*Give P values as exact values whenever suitable.*
☒	☐	For Bayesian analysis, information on the choice of priors and Markov chain Monte Carlo settings
☒	☐	For hierarchical and complex designs, identification of the appropriate level for tests and full reporting of outcomes
☒	☐	Estimates of effect sizes (e.g. Cohen's *d*, Pearson's *r*), indicating how they were calculated

Our web collection on statistics for biologists contains articles on many of the points above.

Software and code

Policy information about availability of computer code

Data collection	Data was collected using commercially-available software: MetaMorph (), pCLAMP (version 10), and BioSigRP (version 4.4).
Data analysis	Changes of the widths or diameters of cells from bright field images were quantified using algorithms in MATLAB (version 8) originally designed for the measurements of the outer hair cell electromotility, described in detail in Frolenkov et al., Biophys J, 1997. These algorithms are archived in Zenodo with the identifier (https://doi.org/10.5281/zenodo.7896132). Other data analysis was performed using ImageJ (versions 1.48 and 1.52), Microsoft Excel (versions 2010, 2013, and 365), GraphPad Prism (versions 6 and 9), Origin (version 9), and Clampfit (version 10).

For manuscripts utilizing custom algorithms or software that are central to the research but not yet described in published literature, software must be made available to editors and reviewers. We strongly encourage code deposition in a community repository (e.g. GitHub). See the Nature Portfolio guidelines for submitting code & software for further information.

Data

Policy information about availability of data

All manuscripts must include a data availability statement. This statement should provide the following information, where applicable:

- Accession codes, unique identifiers, or web links for publicly available datasets
- A description of any restrictions on data availability
- For clinical datasets or third party data, please ensure that the statement adheres to our policy

Source data are provided with this paper. Hearing testing records and all time-lapse and 3D imaging stacks generated during the current study are available from

the corresponding authors on reasonable request.

Human research participants

Policy information about studies involving human research participants and Sex and Gender in Research.

Reporting on sex and gender	N/A
Population characteristics	N/A
Recruitment	N/A
Ethics oversight	N/A

Note that full information on the approval of the study protocol must also be provided in the manuscript.

Field-specific reporting

Please select the one below that is the best fit for your research. If you are not sure, read the appropriate sections before making your selection.

[x] Life sciences   [ ] Behavioural & social sciences   [ ] Ecological, evolutionary & environmental sciences

For a reference copy of the document with all sections, see nature.com/documents/nr-reporting-summary-flat.pdf

Life sciences study design

All studies must disclose on these points even when the disclosure is negative.

Sample size	Initial sample sizes (for pilot experiments) were chosen based on previous experiments performed in the laboratory and/or published literature: for hearing testing see Kwan et al., Neuron, 2006.; for TRPA1 currents in heterologous cells and cochear cells see Stepanyan et al., JARO, 2011; for calcium waves and bright field imaging experiments see Tritsch et al., Nature, 2007. Sample sizes were then recalculated after pilot data was collected. All sample size calculations were performed using a power of 80% and a type 1 error of 0.05.
Data exclusions	Mice that exhibited complications after the intraperitoneal injections of anesthetic (e.g., significant weight loss, infections, or leg paralysis) were removed from the study and humanely euthanized (n=8). Also, mice that exhibited significant hearing loss before any noise exposure were not included in the study (n=3).
Replication	Data showed in the manuscript are the result of several cohorts of mice where the results were successfully replicated. Details on the number of independent experiments performed are provided in the figure legends or the Source Data file.
Randomization	Mice were assigned to experimental groups that were balanced in regards to animal age, weight, and sex.
Blinding	Experiments and data analysis were performed by individuals blind to the mouse genotype.

Reporting for specific materials, systems and methods

We require information from authors about some types of materials, experimental systems and methods used in many studies. Here, indicate whether each material, system or method listed is relevant to your study. If you are not sure if a list item applies to your research, read the appropriate section before selecting a response.

Materials & experimental systems

n/a	Involved in the study	
[ ]	[x]	Antibodies
[ ]	[x]	Eukaryotic cell lines
[x]	[ ]	Palaeontology and archaeology
[ ]	[x]	Animals and other organisms
[x]	[ ]	Clinical data
[x]	[ ]	Dual use research of concern

Methods

n/a	Involved in the study	
[x]	[ ]	ChIP-seq
[x]	[ ]	Flow cytometry
[x]	[ ]	MRI-based neuroimaging

Antibodies

Antibodies used	Mouse anti-hPLAP (clone 8B6, catalog # A2951, lot # 059K4853, Sigma-Aldrich); rabbit anti-HNE (polyclonal, catalog # ab46545, Abcam); mouse anti-CtBP2 (monoclonal, clone 16, catalog # 612044, BD Biosciences); goat anti-mouse IgG2a Alexa Fluor 488

	(polyclonal, catalog # A-21131, ThermoFisher Scientific); goat anti-mouse IgG Alexa Fluor 568 (polyclonal, catalog # A-11004, ThermoFisher Scientific); mouse anti-rabbit IgG Dylight 488 (Jackson Immunoresearch, product has been discontinued); goat anti-mouse IgG1 Alexa Fluor 555 (polyclonal, catalog # A21127, ThermoFisher Scientific).
Validation	According to the manufacturer, the specificity of the Anti-PLAP antibody was tested in SDS gels and it only reacts with hPLAP and not with PLAP-like enzymes. In our experiments, this antibody did not produce any labeling of wild type mouse cochlear tissue, and it only recognized signals in the Trpa1-/- tissue that had the PLAP reporter.<br><br>The anti-HNE antibody has been referenced in 412 publications. It's reactivity is species independent according to the manufaturer. In addition, we tested its performance in cochlear tissue exposed to 3 increasing concentrations of hydrogen peroxide in vitro.<br><br>Anti-CtBP2 has been validated in CtBP2/RIBEYE knockout mice in Becker et al., Elife, 2018.

Eukaryotic cell lines

Policy information about cell lines and Sex and Gender in Research

Cell line source(s)	HEK293 cells were obtained from ATCC.
Authentication	Cell lines were not authenticated
Mycoplasma contamination	Cell lines were not tested for mycoplasma contamination
Commonly misidentified lines<br>(See ICLAC register)	No commonly misidentified lines were used

Animals and other research organisms

Policy information about studies involving animals; ARRIVE guidelines recommended for reporting animal research, and Sex and Gender in Research

Laboratory animals	We used TRPA1-deficient mice () that were backcrossed to C57Bl/6 mice for 10 generations, and sibling breeding pairs were established. We used Trpa1+/+ and Trpa1-/- mice at early postnatal days (P0 to P8), and young adults (P18-19, and 3-4 weeks old).
Wild animals	The study did not involve wild animals
Reporting on sex	Both female and male mice were included in this study. All experimental cohorts roughly had a 50/50 gender distribution and no significant differences were observed between genders.
Field-collected samples	The study did not involve field-collected samples
Ethics oversight	All animal procedures were approved by the University of Kentucky Animal Care and Use Committee (protocols 00903M2005, 2019-3414 and 2020-3535).

Note that full information on the approval of the study protocol must also be provided in the manuscript.
